# Turbopropellerlappen zur Rekonstruktion eines Ellenbogendefektes in Regionalanästhesie

**DOI:** 10.1007/s00113-022-01193-w

**Published:** 2022-05-25

**Authors:** Andrej Ring, Martin Bauer, Niklas-Chris Dellmann, Sebastian Ulrich Bushart, Mathias Witt

**Affiliations:** 1 Klinik für Plastische Chirurgie, SLG St. Paulus GmbH, St. Rochus Hospital, Glückaufstr. 10, 44575 Castrop-Rauxel, Deutschland; 2 Klinik für Anästhesiologie, Intensiv- und Schmerzmedizin, SLG St. Paulus GmbH, St. Rochus Hospital, Castrop-Rauxel, Deutschland

**Keywords:** Perforator, Lappenplastik, Olecranon, Mikrochirurgie, Plexus brachialis, Perforator, Defect coverage, Olecranon, Microsurgery, Brachial plexus

## Abstract

Eine alternative Methode zur plastischen Defektdeckung am Ellenbogen wird vorgestellt. Verwendet wurde ein perforatorbasierter, retrograd gestielter Propellerlappen vom lateralen Oberarm mit additiver mikrovaskulärer „Turbo“-Anastomosierung an die A. und V. radialis. Die Turbolappenplastik wurde in Regionalanästhesie durchgeführt.

## Abstract (en)

A method for the reconstruction of an extensive soft tissue defect after a complicating olecranon fracture is presented. A perforator-based retrograde pedicled propeller flap was used from the lateral upper arm with additive microvascular “turbo”-anastomosis to the radial artery and vein. The turbo-flap was performed under regional anesthesia.

## Anamnese

**Figure 1:**
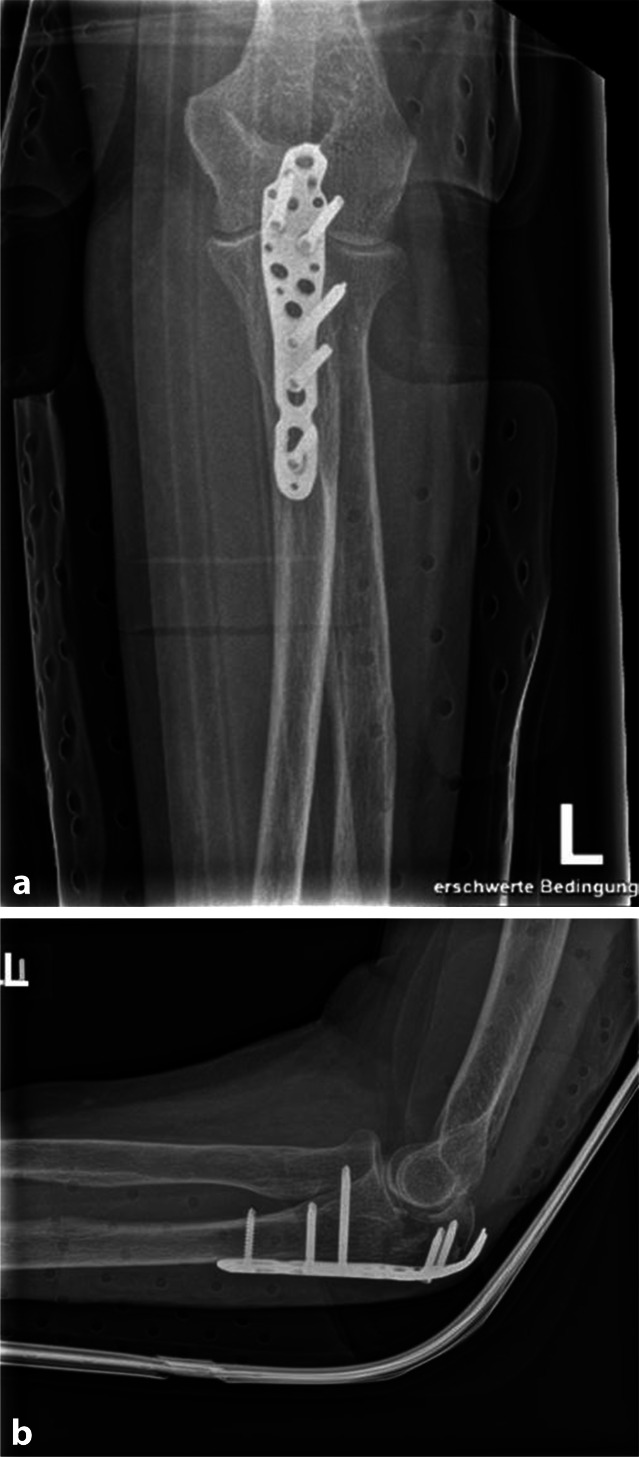

Eine 85-jährige Patientin zog sich nach einem Sturz in häuslicher Umgebung eine Mehrfragmentfraktur des linken Olecranons zu. Es erfolgte eine offene Reposition und Osteosynthese mittels winkelstabiler anatomischer Olecranonplatte (Abb. 1a, b). Drei Wochen postoperativ entwickelte sich eine Wundheilungsstörung, welche eine Wundrevision mit Metallentfernung bei Plattenlagerinfekt notwendig machte.

An Nebendiagnosen bestand eine Tachyarrhythmie bei Vorhofflimmern mit mehrjähriger Einnahme von Verapamil und Apixaban. Des Weiteren bestand ein Zustand nach Stent-Versorgung bei Stenose der linken A. femoralis superficialis. Zudem wurde 5 Tage nach der Frakturversorgung bei neu aufgetretener Schwellung und Schmerzhaftigkeit des linken Beines eine Thrombose des tiefen Beinvenensystems in Becken‑, Leiste‑, Oberschenkel‑, Popliteal- und Unterschenkeletage (4-Etagen-TVT) mit Lungenarterienembolie festgestellt.

## Befund und Diagnose

**Figure 2:**
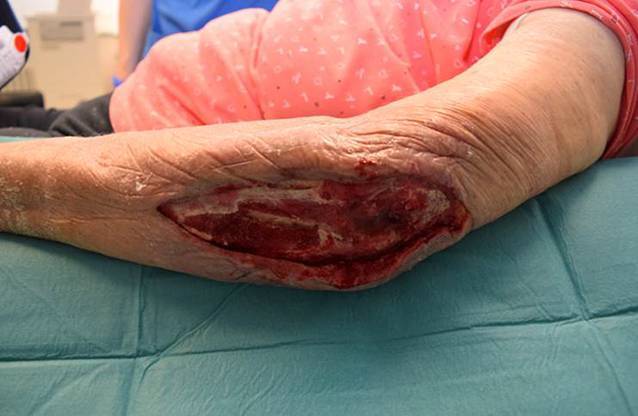

Nach erfolgter Metallentfernung im Rahmen der radikalen Wundrevision und Versorgung des entstandenen Defektes über dem linken Olecranon mit einem Vakuumversiegelungsverband erfolgte die Kontaktaufnahme zur plastischen Deckung des ausgedehnten (6 × 16 cm) Haut-Weichteil-Defektes bei Exposition der nichtkonsolidierten Frakturzone (Abb. 2).

## Therapie und Verlauf

Da die Patientin eine Allgemeinanästhesie kategorisch ablehnte, wurde die einzeitige Rekonstruktion ausschließlich unter Verwendung von regionalem Gewebe der verletzten Extremität in interskalenärer Plexusblockade durchgeführt. Zur Planung des Gewebetransfers wurde eine Power-Doppler-Sonographie der perforatorversorgten Territorien am lateralen Oberarm durchgeführt. Hierbei konnten eine erhaltene arterielle Versorgung des defektnahen Weichteilmantels über die Aa. recurrens radialis et collateralis radialis sowie eine territoriale Versorgung der proximalen Region des lateralen Oberarms über die A. circumflexa anterior humeri bestätigt werden. Die Lappenplastik wurde somit als „dual axis perforator flap“ mit doppelter axialer Gefäßversorgung konzipiert.

**Figure 3:**
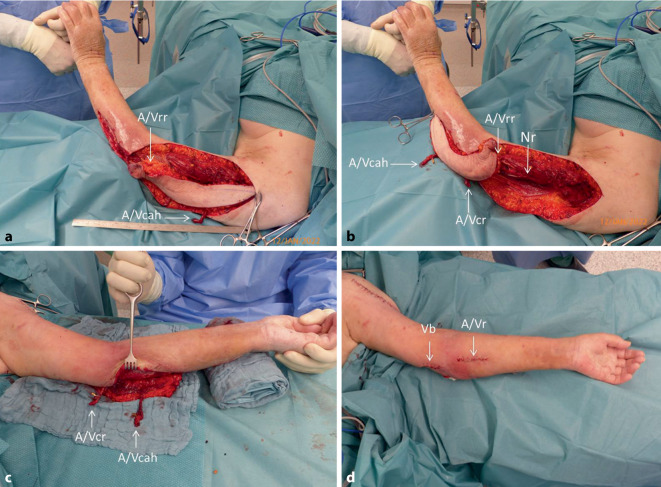

Nach einem anfrischenden Débridement des Wundgrundes und einer Gelenkspülung wurde ein fasziokutaner, distal an A. und V. recurrens radialis (A/Vrr) gestielter, retrograd perfundierter Lappen unter Dissektion des N. radialis (Nr) und interseptaler Präparation der A. und V. collateralis radialis (A/Vcr) gehoben. Im proximalen Bereich des Oberarmlappen wurden die A. und. V. circumflexa anterior humeri (A/Vcah) für die spätere additive mikrochirurgische „Turbo“-Anastomosierung des proximalen Lappenterritoriums präpariert. Der Lappen wurde anschließend um 180 Grad um den distalen Perforator in den Defekt am Ellenbogen und am proximalen Unterarm im Uhrzeigersinn gedreht (Abb. 3a, b).

Nach der Präparation der Anschlussstelle für die additive arterielle und venöse Anastomose wurden über einen getrennten Zugang am volaren Unterarm die A. und V. radialis dargestellt. Der Perforatorstiel mit A. und V. circumflexa anterior humeri (A/Vcah) wurde nach subkutaner Tunnelung arteriell End-zu-Seit und venös End-zu-End an die A. und V. radialis (A/Vr) anastomosiert. Über einen weiteren Zugang auf der Höhe der Ellenbeuge wurde nach subkutaner Tunnelung die V. collateralis radialis an einen Zufluss der V. basilica (Vb) mittels 3,0 mm Coupler-Ring anastomosiert. Anschließend wurde der Propellerlappen spannungsfrei in den Defekt eingepasst und der Hebedefekt am lateralen Oberarm primär verschlossen (Abb. 3c, d). Die präoperativ vorbestandene Antikoagulation mit niedermolekularem Heparin (Enoxaparin-Natrium) im therapeutischen Bereich wurde fortgeführt.

**Figure 4:**
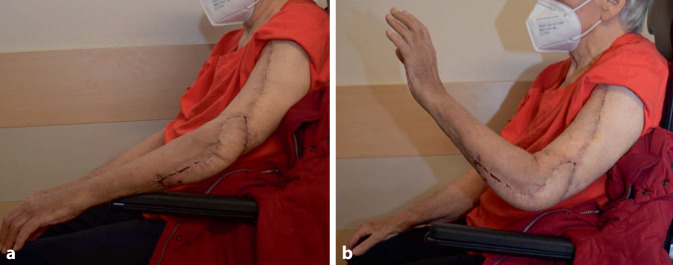

Eine Ruhigstellung in einer gut wattegepolsterter Schiene wurde bis zur Wundkonsolidierung fortgeführt und ab dem 10. postoperativen Tag nach Rücksprache mit unfallchirurgischen Kollegen mit der Intensivierung der Beübung des Ellenbogengelenkes begonnen. Die Wundheilung verlief per primam. Eine aktive Beweglichkeit mit Ausmaßen von 10–90 Grad für Streckung und Beugung bei Schmerzfreiheit bestand 3 Wochen nach der Rekonstruktion (Abb. 4a, b).

## Diskussion

Weichteildefekte am Ellenbogen bedürfen einer Wiederherstellung mit möglichst dünnem und gleichzeitig widerstandsfähigem Gewebe. Unterschiedliche Verfahren, einschließlich freier Spalthauttransplantation, lokaler und regionaler Lappenplastiken wie z. B. Radialislappen und lateraler Oberarmlappen als auch gestielte Fernlappenplastiken wie der Latissimus-dorsi-Lappen und eine Reihe an freien Lappenplastiken, können für die Rekonstruktion verwendet werden.

Grundsätzlich sollten bei der Auswahl des rekonstruktiven Verfahrens die individuellen Anforderungen berücksichtigt werden. Dabei muss dem Allgemeinzustand des Patienten, seiner Mobilität und seiner perioperativen Belastungsfähigkeit Rechnung getragen werden. Auch spielen die Compliance des Patienten, die Durchblutungssituation an der Extremität, die Morbidität der Spenderregion und die zu erwartenden Bewegungsausmaße im betroffenen Gelenk eine wesentliche Rolle. Ebenso können weitere geplante Revisions- und Korrektureingriffe am verletzten Gelenk die initiale Auswahl rekonstruktiver Verfahren beeinflussen [1].

Der Erfolg traditioneller „Mono-Axis-Perforator“-Lappenplastiken ist durch die Größe eines Perforasoms bestimmt. Häufig ist die perfundierte Lappenspitze der kritische Faktor bei der plastisch-rekonstruktiven Defektdeckung [2].

Die Techniken des retrograd gestielten als „reverse flow“ bekannten Lappens aus der lateralen Oberarmregion als auch die der perforatorbasierten Propellerlappenplastik wurden für Rekonstruktionen von Weichteildefekten im Ellenbogenbereich entwickelt und stellen nach wie vor eine exzellente lokale Option dar [3–5]. Wird in solchen Fällen jedoch ein größerer („extended“) Lappen gebraucht, so wird von den meisten Autoren ein 2‑zeitiges Vorgehen mit einer Präkonditionierung des Lappen („delay“) empfohlen [4, 5].

In dem hier vorgestellten Fall wurde auf eine wochenlange Präkonditionierung verzichtet.

Stattdessen wurde die Methode einer additiven mikrochirurgischen „Turbo“-Anastomosierung, wie von Semple [6] vorgestellt, gewählt. Hierdurch kann sowohl die arterielle Versorgung als auch die venöse Drainage des Lappens verbessert werden.

In Anlehnung an die Angiosom-Theorie von Taylor und Palmer [7] sowie das Perforasom-Konzept von Saint-Cyr et al. [8] konnte somit praktisch bestätigt werden, dass der Lappenerhalt durch eine vaskuläre Vereinigung angrenzender Perforasome sicherer gestaltet bzw. ein einzeitiges rekonstruktives Vorgehen überhaupt erst ermöglicht werden kann.

Hierfür wurde präoperativ zur sicheren Planung einer ausreichenden Vaskularität und des Designs des für die Rekonstruktion verwendeten Lappens, in Anlehnung an Daigeler et al. [9], die Technik der Power-Doppler-Sonographie zwecks Vereinigung der perforatorversorgten Territorien am lateralen Oberarm erfolgreich eingesetzt.

Die Länge des Lappen wurde in dem hier vorgestellten Fall entsprechend der longitudinalen Defektausdehnung von 16 cm geplant. Dies hat aber zur Folge gehabt, dass der „überlange“ Lappen zwar aus 2 benachbarten Territorien bestand, diese jedoch eine getrennte arterielle Versorgung als auch venöse Drainage aufwiesen. Um das Risiko einer arteriellen Minderdurchblutung als auch einer venösen Stauung des proximalen Lappenterritoriums nach der 180°-Drehung zu reduzieren, wurde das Turbokonzept mit Anlage von additiven venösen als auch arteriellen Supercharged-Anastomosen angewandt.

Der Vorteil der verwendeten Lappenplastik wird von den Autoren in seiner doppelten Gefäßversorgung gesehen. Durch die zusätzlich zu der retrograden gefäßgestielten Versorgung des Lappens angelegten mikrochirurgischen „Turbo“-Anastomosen konnte das Problem der Minderperfusion der letzten Wiese der Lappenplastik gelöst werden. Aufgrund einer derartigen Doppelversorgung des Lappens ergab sich ein weiterer Vorteil dahingehend, dass eine Perfusion des Turbopropellerlappens in jeder Position des Ellenbogens sichergestellt und somit keine Ruhigstellung erforderlich war.

Das hier angewandte Prinzip einer doppelten axialen Gefäßversorgung unter mikrochirurgischer Anlage additiver „Turbo“-Anastomosen kann unter Berücksichtigung anatomischer Gegebenheiten für unterschiedliche Lappenplastiken angewandt werden. Die vorgestellte Methode stellt eine sinnvolle und sichere Erweiterung des klinisch relevanten Perforasom-Konzeptes dar.
